# The extracellular matrix of green algae

**DOI:** 10.1093/plphys/kiad384

**Published:** 2023-07-03

**Authors:** David S Domozych, Josephine G LoRicco

**Affiliations:** Department of Biology, Skidmore College, Saratoga Springs, NY 12866, USA; Department of Biology, Skidmore College, Saratoga Springs, NY 12866, USA

## Abstract

Green algae display a wide range of extracellular matrix (ECM) components that include various types of cell walls (CW), scales, crystalline glycoprotein coverings, hydrophobic compounds, and complex gels or mucilage. Recently, new information derived from genomic/transcriptomic screening, advanced biochemical analyses, immunocytochemical studies, and ecophysiology has significantly enhanced and refined our understanding of the green algal ECM. In the later diverging charophyte group of green algae, the CW and other ECM components provide insight into the evolution of plants and the ways the ECM modulates during environmental stress. Chlorophytes produce diverse ECM components, many of which have been exploited for various uses in medicine, food, and biofuel production. This review highlights major advances in ECM studies of green algae.

**Advances Box**

- Expanded sequencing and mining of genomes and transcriptomes of diverse green algal taxa has provided key insight into the biosynthesis and modulation of ECM components as well as evolutionary links to land plants.
- Synthesis of field investigations and ecophysiology of specific taxa with molecular and biochemical studies has elucidated changes in ECM structure and dynamics during environmental stress.
- Enhanced exploration of green algal ECM for applied uses in food, medicine, nutraceutical, and biofuel industries has catalyzed advanced biochemical analyses of the ECM.
- Application of novel immunochemical and microscopy-based technologies has shed light on the complex structural designs and development of ECM components.

**Outstanding Questions Box**

- What is the composition of the CW proteome and what are the functions of proteins, including AGP, extensin, and expansin, of green algae?
- What are the cytokinetic mechanisms involved in the ZCC clade of charophytes, including furrowing and cell plate/phragmoplast formation, and the CW polymers required for completion of cytokinesis?
- What are the biosynthetic pathways, secretion dynamics, and functional roles of hydrophobic components of the ECM (e.g. algaenan, sporopollenin, phenolics, lipid coverings) of green algae?
- How do the ECMs modulate in response to abiotic and biotic stress, and what are the roles of specific components such as sulfated polysaccharides and EPS gels?
- What culturing, harvesting, and processing technologies will be optimal for efficacious use of green algae and their ECMs in the food, medicine, biofuels, and other industries?

## Introduction

**Figure 1.**
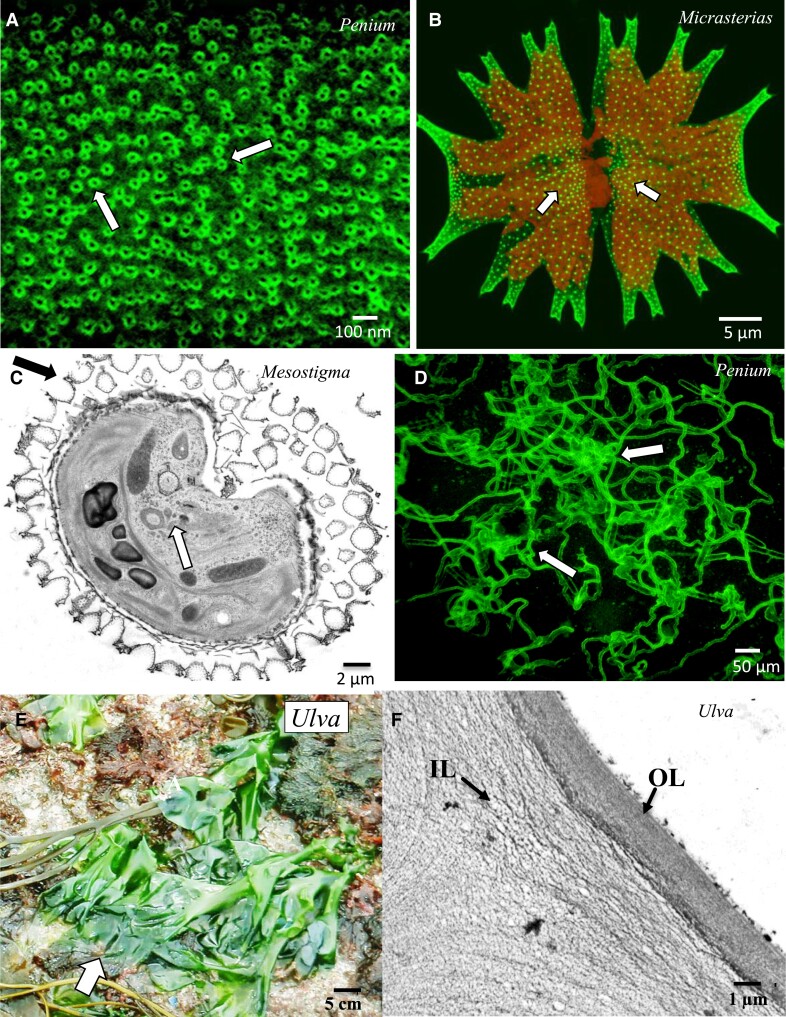
Examples of ECM diversity in green algae. **A)** The late-divergent charophyte *Penium margaritaceum* possesses a unique Ca^2+^complex pectin lattice (arrows) in its CW. Labeled with the HG-binding mAb JIM5. Fluorescence light microscopy (FLM). **B)** The CW of the late-divergent charophyte *Micrasterias radiata* possesses pores labeled with the AGP-specific mAb JIM13 (arrows, FLM). **C)** In the early-divergent charophyte *Mesostigma viridie*, multiple layers of scales cover the surface of motile cells (arrow). TEM image. **D**) The gliding trails of the EPS of *P. margaritaceum* (arrows). Labeled with 0.75 fluorescent beads (FLM). **E)** The Ulvophyte *Ulva* (arrow) is a common coastal seaweed whose CW polysaccharides have been extracted for multiple uses. **F)** The CW consists of an inner (IL) and outer (OL) layer and consists of cellulose, XyG, glucuronan, and the sulfated polysaccharide, ulvan (TEM).

Green algae comprise a diverse assemblage of organisms that are part of the green lineage (Chloroplastida) of the Archaeplastida (i.e. green algae and plants; Adl et al. 2019; Bowles et al. 2022). These algae arose over 1 billion years ago, and their extant taxa now occupy most photic habitats of the planet, where they play major roles in the dynamics of the atmosphere, biogeochemistry, and food chains. Over 500 million years ago, an ancestor of the modern-day green algal group, the Zygnematophyceae (Charophyta or Streptophyta; Delwiche and Cooper 2015; Rensing 2020; Schreiber et al. 2022), successfully transitioned from a freshwater to a terrestrial habitat and ultimately gave rise to land plants, an event that profoundly changed the natural history of the planet (de Vries and Archiblad 2018; Fürst-Jansen et al. 2020; Permann et al. 2022). Green algae display a truly diverse range of phenotypes that include unicellular picoplankton (e.g. *Ostreococcus*), unicellular and colonial flagellates (e.g. *Chlamydomonas*, *Volvox*), filaments (e.g. *Ulothrix*, *Spirogyra*), coenocytic siphons (e.g. *Acetabularia*), and distinct 3-dimensional thalli (e.g. *Ulva*, *Chara*, *Coleochaete*). Green algae are major primary producers of marine, freshwater, and terrestrial habitats, may form spectacular blooms, are common residents of aquatic biofilms and may even form unique symbiotic associations with fungi (e.g. lichens) and various invertebrates. Central to their structure, development, physiology, and evolution is an extracellular matrix (ECM) that may manifest as a cell wall (CW), crystalline glycoprotein coverings, layers of scales, hydrophobic compounds, and/or a gel-like, extracellular polymeric substances (EPS) (Fig. 1). Green algal ECMs have long been studied by plant biologists (for reviews, see Popper et al. 2011; Domozych et al. 2012), and recently, detailed data acquired using molecular biology, immunology, high-resolution microscopy imaging, and ecophysiology have provided novel insight into our understanding of their structural architecture, biosynthesis, and stress-related modulations. Likewise, studies of green algae and their ECMs have been fueled by their multiple uses in human economy (e.g. food, medicines; Mišurcová et al. 2012; Roleda and Heesch 2021; Li et al. 2022) and, most recently, as sources of degradable biomass for production of biofuels (Soni et al. 2021). This update highlights recent major advances in ECM biology of the green algae.

### The ECM of green algae: an overview

Green algae make up an estimated 22,000 extant species (Guiry 2012) that are currently classified in 3 phyla (Leliaert et al. 2012; Leliaert 2019; Li et al. 2020): (1) Streptophyta (also called Charophyta or basal Streptophyta; Mattox and Stewart 1984; Becker and Marin 2009); (2) Prasinodermophyta; and (3) Chlorophyta. It is important to note that our understanding of the taxonomy and phylogeny of green algae is continuously evolving as more information derived from genomic/transcriptomic sequencing and enhanced screening of more diverse assemblages of taxa have become available. For more up-to-date and detailed information, readers should consult Leliaert (2019), Li et al. (2021), Bachy et al. (2022), and Hess et al. (2022). Likewise, Table 1 lists those green algae whose genome/transcriptome have been investigated and may serve as valuable resources for elucidating multiple aspects of green algal biology.

**Table 1.** Genome and transcriptome resources for ECM studies of green algae

*Streptophyta/Charophyta*			
Charophytes	*Mesostigma viride*	Wang et al. (2020) Liang et al. (2019)	doi: 10.1038/s41477-019-0560-3 doi: 10.1002/advs.201901850
	Chlorokybus atmophyticus	Irisarri et al. (2021) Wang et al. (2020)	doi: 10.1098/rspb.2021.2168 doi: 10.1038/s41477-019-0560-3
	*Klebsormidium flaccidum* *(now Klebsormidium nitens)*	Hori et al. (2014)	doi: 10.1038/ncomms4978
	*Chara braunii*	Nishiyama et al. (2018)	doi: 10.1016/j.cell.2018.06033
	*Spirogloea muscicola*	Cheng et al. (2019a, b)	doi: 1016/j.cell.2019.019
	*Mesotaenium endlicherianum*	Cheng et al. (2019a, b)	doi: 1016/j.cell.2019.019
	*Penium margaritaceum*	Jiao et al. (2020)	doi: 10.1016/j.cell.2020.04.019
	*Spirogyra pratensis*	Van de Poel et al. (2016) “One thousand transcriptomes” (2019)	doi: 1104/pp.16.00299 doi: 1038/s41586-019-1693-2
	*Zygnema sp*.	Feng et al. (2023)	doi: 10.1101/2023.01.31.526407
Prasinodermophyta			
	*Prasinoderma colonial*	Li et al. (2020)	doi: 10.1038/s41559-020-1221-7
***Chlorophyta***			
Prasinophytes	*Ostreococcus*- 3 species	Blanc-Mathieu et al. (2014) Palenick et al. (2007)	doi: 10.1186/1471-2164-15-1130 doi: 10.1073/pnas.0611046104
	*Pyraminonas parkeae*	Satjarak and Graham (2017)	doi: 10.1111/jpy.12566
	*Micromonas sp*.	Delmont et al. (2015) Worden et al. (2009)	doi: 10.3389/fmicb.2015.01090 doi: 10.1126/science.1167222
	*Tetraselmis suecica* ^a^	Lauritano et al. (2019)	doi: 10.1038/s41598-019-39860-5
	*Bathycoccus prasinos*	Moreau et al. (2012)	doi: 10.1186/gb-2012-13-8-r74
Ulvophytes	*Ulva mutabilis*	De Clerck et al. (2018)	doi: 10.1016/j.cub.2018.08.015
	*Ulva prolifera*	He et al. (2021)	doi: 10.1007/s00343-020-0212-5
	*Ostreobuim quekettii*	Iha et al. (2021)	doi: 10.1016/j.cub.2021.01.018
	*Caulerpa lentillifera*	Arimoto et al. (2019)	doi: 10.1093/dnares/dsz002
	*Caulerpa taxifolia* ^a^	Ranjan et al. (2015)	doi: 10.1371/journal.pgen.1004900
Trebouxiophyceae	*Chlorella variabilis*	Blanc et al. (2010)	doi: 10.1105/tpc.110.076406
	*Chlorella sorokiniana*	Arriola et al. (2017)	doi: 10.1111/tpj.13789
	*Botryococcus braunii*	Browne et al. (2017)	doi: 10.1128/genomeA.00215-17
Chlorophyceae	*Dunaliella salina*	Polle et al. (2017)	doi: 10.1128/genomeA.01105-17
	*Chlamydomonas reinhardtii*	Merchant et al. (2007)	doi: 10.1126/science.1143609
	*Chlamydomonas eustigma*	Hirooka et al. (2017)	doi: 10.1073/pnas.1707072114
	*Scenedesmus glucoliberatum*	Mancipe et al. (2021)	doi: 10.1111/jam.15311
	*Scenedesmus sp*.	Nelson et al. (2019) Soos et al. (2021)	doi: 10.1016/j.isci.2018.12.035 doi: 10.1016/j.algal.2020.102181
	*Scenedesmus obliquus*	Starkenburg et al. (2017) Chen et al. (2020)	doi: 10.1128/genomeA.00617-17 doi: 10.1186/s12864-020-07142-4
	*Volvox carteri*	Prochnik et al. (2010)	doi: 10.1126/science.1188800
	*Astrephomene*	Yamashita et al. (2021)	doi: 10.1038/s41598-021-01521-x

^a^Indicates only transcriptome data available.

**Figure 2.**
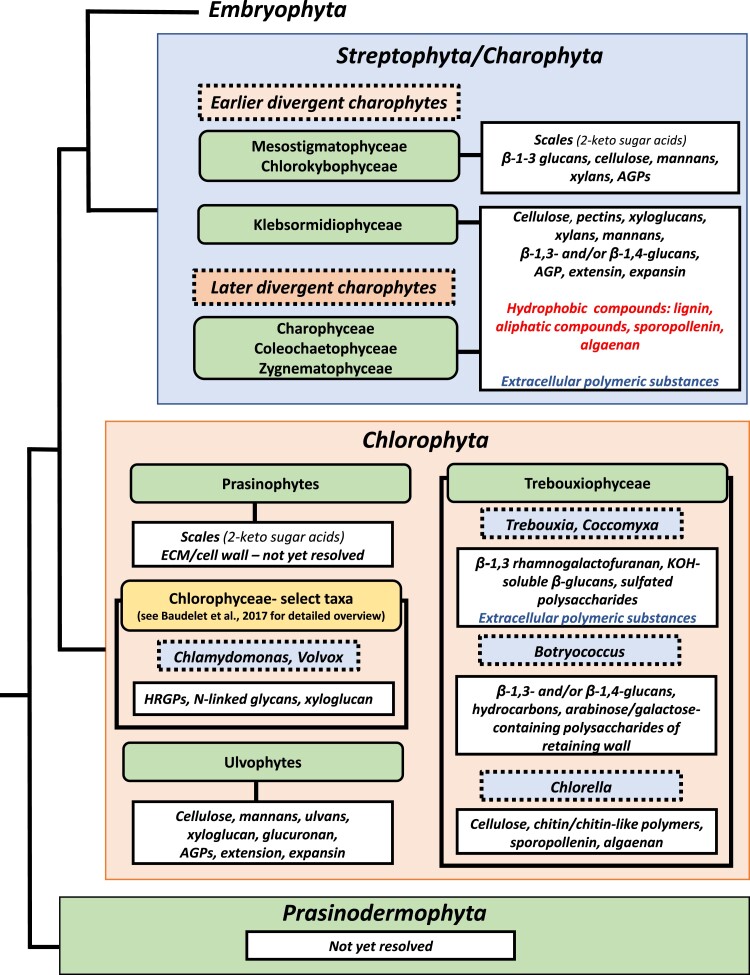
Summary of major ECM components found in the major groups of green algae.

Much of what is known about the ECMs of green algae is based on biochemical, cellular, and molecular studies of small samples of select taxa. It is clear that we are only in an infancy stage of fully understanding this great diversity in ECM structures, their functions, and their development. Yet, what we do know paints a fascinating picture of how ECMs are critical to survival in vastly different ecosystems, modulations in response to environmental stress, and the evolution of modern-day green algae and plants. This review describes 5 major ECM types found in green algae based on both their structural/biochemical features (Text Box 1) and taxonomic/phylogenetic relationships (Fig. 2). These include scale coverings, glycoprotein coverings, hydrophobic components, EPS, and CWs. Although it is recognized that this grouping of ECM types is somewhat artificial, it provides a basis for our current understanding of the green algal ECM and a stimulus for future research.

**Text Box 1**

**The ECM components of the green algae**

The ECM components of green algae can be loosely classified into 5 major types:

1. Scale coverings are commonly found on the plasma membrane/flagellar membranes of some unicellular taxa (e.g. prasinophytes and the charophyte, *Mesostigma*) and motile zoospores and sperm cells (e.g. the charophytes, *Chara*, *Coleochaete*).
2. Glycoprotein coverings that are highlighted by large amounts of the amino acid hydroxyproline are found in several chlorophyte taxa, most notably in unicellular and colonial flagellates including *Chlamydomonas* and *Volvox*.
3. Hydrophobic ECM components that include sporopollenin, algaenan, and lipid-like cuticle-like compounds have been identified throughout the chlorophytes and charophytes, including resting cell and spore coatings.
4. EPS consists of highly complex polysaccharide and protein components secreted beyond the CWs that often form gels/mucilages are found throughout the green algae.
5. CWs consisting of a framework of polysaccharide fibrils (e.g. cellulose, mannans, xylans) that are embedded in a matrix of polysaccharides and glycosylated proteins are exhibited in charophytes and chlorophytes.

#### Scales

Samples taken from the photic planktonic zones of most ocean waters will often contain prasinophytes. The Prasinophyceae comprise a paraphyletic assemblage of unicellular, mostly marine organisms that are believed to be the earliest divergent Chlorophyta (Bachy et al. 2022). Many taxa are distinguished by the presence of 1 or more layers of scales attached to the plasma membrane and/or flagellar membrane (Moestrup and Walne 1979; Perasso et al. 2000). Scale chemistry is highlighted by the 2-keto sugar acids, deoxy-5-*O*-methyl-*manno*-2-octulosonic acid and deoxy-*lyxo*-2-heptulosaric acid (Becker et al. 1994). The various shaped and sized scales are synthesized in the Golgi apparatus and are subsequently released onto the cell surface in a process that may include a vacuole-like scale reservoir. The 2-keto sugar acids found in scales are also found in the thecal covering of the chlorophyte, *Tetraselmis* (Becker et al. 1998; Kermanshahi-Pour et al. 2014) and in the spectacular basket scales of the early divergent charophyte *Mesostigma viride* (Domozych et al. 1991). Recently, biosynthesis and transport genes for KdO were identified in *Mesostigma* (Liang et al. 2019). It is also important to note that the zoospores and sperm cells of many green algae, including the charophytes *Chlorokybus*, *Coleochaete*, and *Chara*, contain scales, but their chemistry and functional roles are presently unknown.

A long-standing question concerning prasinophytes is whether they produce ECM components other than scales during different life cycle stages. Although no biochemical data are currently available, molecular studies have provided some initial insight (Worden et al. 2009; Blanc-Mathieu et al. 2014; Satjarak and Graham 2017). In a screening of the *Pyramimonas parkeae* genome for carbohydrate-active enzymes (CAZYmes), sequences that cluster with bacterial genes that encode cellulose synthases, including regions coding for domains of plant cellulose synthases, were identified (Satjarak and Graham 2017). Genes involved in pectin and xyloglucan (XyG) processing were also identified, including putative galacturonosyl-transferases, pectin acetyl esterases, pectin-degrading enzymes, and ß-galactosidase and xylosidase.

#### Glycoprotein coverings

*Chlamydomonas*, *Volvox*, and other related flagellates are commonly encountered chlorophytes of aquatic and terrestrial ecosystems. The ECMs of these organisms are comprised of distinct, hydroxyproline-rich glycoproteins (HRGP) that form a thin CW-like covering and, in colonial forms, an extensive matrix that holds the cells in place in the colony. The CW of the unicellular *Chlamydomonas reinhardtii* consists of an insoluble framework and about 20 hydroxyproline (Hyp)-containing, chaotrope-soluble glycoproteins (Lee et al. 2007; Voight et al. 2010; Barolo et al. 2022). A detailed, bioinformatics-based comparison of *Chlamydomonas* CW HRGPs with plant extensins may be found in Liu et al. (2016). Recently, a CW integrity–monitoring mechanism has been described in *Chlamydomonas* that senses osmotic stress and mechanical defects of its CWs and subsequently regulates CW-gene expression (Cronmiller et al. 2019). The colonial *Volvox* ECM consists of a diverse array of HRGPs (Sumper and Hallmann 1998). Its ECM matrix contains specific cell-type HRGPs, the pherophorins, that are central to the developmental dynamics of the colony (Hallmann 2006; von der Heyde and Hallmann 2022). In addition to the HRGP components of the ECM, *Chlamydomonas* also secretes complex N-linked glycans. Xylosyltransferases that heterogeneously xylosylate these glycans were recently described (Lucas et al. 2021). Elucidation of the biochemistry, genetics, and development of the ECMs of *Chlamydomonas* and *Volvox* has been critical in that they are central to understanding the biology of these valuable model organisms employed in studies of algal/plant stress biology, sexual reproduction, photosynthesis, organelle biosynthesis, circadian rhythms, and phototaxis (Matt and Umen 2016; Salomé and Merchant 2019; Umen 2020).

#### Hydrophobic components: Sporopollenin, algaenan, lignin, cuticles

The ECM of diverse taxa of the Chlorophyta and Charophyta possess hydrophobic components, including sporopollenin, algaenan, lignin, and other molecules. Sporopollenin is a highly resistant and complex heteropolymer partly composed of hydroxylated polyketides (Li et al. 2019) that is commonly found in the outer walls of pollen grains and land plant spores. Algaenan is a nonhydrolyzable aliphatic biopolymer composed of long-chain, even carbon–numbered, *ω*9-unsaturated *ω*-hydroxy fatty acid monomers varying in chain length from 30 to 34 carbon atoms (Rodrigues and de Silva Bon 2011). These are often ester-linked to form long, highly resistant polymers. Algaenan and sporopollenin have been identified in the spore walls of some charophytes (Niklas et al. 2017; de Vries and Archibald 2018; Permann et al. 2021a) and in the CWs of chlorophytes (Rodrigues and de Silva Bon 2011; Bachy et al. 2022). Polyketide synthase (PKS) and anther-specific chalcone synthase-like (ASCL) are fundamental players in the biosynthetic pathway of hydroxylated polyketides that serve as the precursors for sporopollenin (Suh and Ashton 2022). Screening of the genomes of the zygnematophytes *Spirogloea muscicola*, *Mesotaenium endlicherianum* (Cheng et al. 2019a, 2019b), and *Penium margaritaceum* (Jaio et al. 2020) has revealed putative PKSs, but they do not possess the recognized ASCL-specific active site residues (Suh and Ashton 2022).

Various phenolic compounds have been identified in charophytes (de Vries et al. 2017), including lignin in the CWs of later-divergent taxa (Sørensen et al. 2010, 2011). These and other phenolic compounds are products of the phenylpropanoid pathway (Rieseberg et al. 2023). Homologs of phenylalanine lyase, a key enzyme in phenylpropanoid synthesis, and other core phenylpropanoid biosynthetic genes have been identified in *Klebsormidium nitens* and *Chara braunii* (de Vries et al. 2017; Rieseberg et al. 2023) but were not found in *P. margaritaceum* (Jaio et al. 2020). This suggests a complex evolutionary history in the production of soluble lignin-like compounds, which are deposited into the CW of some charophytes.

Many phenolic compounds are employed in plants to enhance the structure and defense of the CW from abiotic and biotic stress, act as antioxidants, protect against UV radiation, and contribute to the regulation of water retention, for example, homoiohydry (Popper et al. 2011; de Vries and Archibald 2018; Kumar et al. 2020). For example, waterproof aromatic substances were detected by RAMAN spectroscopy in zygospores of some zygnematophytes (e.g. Permann et al. 2021a, 2021b; Permann et al. 2022). An array of phenolics (e.g. tannin-like compounds, flavonoids) have also been isolated and characterized in several charophytes (Aigner et al. 2013; Pichrtová et al. 2013; Permann et al. 2022). These studies have also described their importance in the ecophysiology of certain charophytes (e.g. *Zygogonium*) as well as in the overall health of glaciers (Dial et al. 2018; Davies et al. 2022). Most of these phenolics have been located in vacuoles or are released beyond the ECM. However, the discovery of an EPS-based “sunscreen” in the zygnematophyte *Serritaenia* (Busch and Hess (2022) that acts as a photoprotective and whose production is stimulated by UVB light suggests that some are closely associated with the ECM.

Recent interest has emerged as to the putative presence of aliphatic polymers in the ECM of some charophytes and their implications on the evolutionary origin of hydrophobic coverings in land plants (e.g. cutin, suberin), including the cuticle of epidermal cells and the endodermis of roots (Niklas et al. 2017; Philippe et al. 2020). Kondo et al. (2016) described a cuticle-like hydrophobic layer composed of lipids and glycoproteins in the ECM of *Klebsormidium flaccidum*. The lipid constituents of this layer, though, were notably different from the cutin polymers typically found in land plant cuticles. Screening of the *P. margaritaceum* genome and those of other late-divergent charophytes (Jiao et al. 2020) revealed homologs of genes for cuticle biosynthetic, transport, assembly frameworks (e.g. cutin synthase and BODYGUARD) that are known in *Arabidopsis thaliana*. However, other gene families involved in cuticle formation in land plants were not identified. It was concluded that phylogenetic distribution of these genes is consistent with stepwise expansion and neofunctionalization of ancient core lipid biosynthetic machinery to synthesize structural lipid precursors.

#### EPS: a future challenge for glycomics and proteomics

Many green algae produce an EPS that is secreted beyond their CWs (Boney 1981; Gerrath 1993; Domozych and Bagdan 2022). In many taxa, especially charophytes (e.g. zygnematophytes), the EPS manifests in distinct gel-like mucilages that surround the cell. EPS is produced by both planktonic and sessile green taxa and in certain cases, in very large amounts (e.g. massive amounts of EPS during spring ephemeral blooms by filamentous zygnematophytes; Domozych and Domozych 2007). EPS has multiple diverse functions in charophytes, including water retention, buoyancy, gliding motility, sexual reproduction, and contribution to the matrix of biofilms. In those charophytes whose EPS has been analyzed, highly-branched anionic polysaccharides with large amounts of fucose, galactose (Gal), and glucuronic acid (GlcA), with variable levels of sulfate and with associated proteins, have been described (Kiemle et al. 2007; Domozych and Bagdan 2022).

Many chlorophytes also secrete EPS beyond the cell. For example, some *Chlorella* species produce a large and complex ECM beyond the CW that provides herbicide and antibiotic resistance (Nazos et al. 2020). Spribille et al. (2020) described an “extracellular interaction matrix,” that is, a gelatinous-like zone beyond the CWs, that harbors nonphotosynthesizing organisms that are not traditionally recognized as lichen symbionts. EPS has also been examined as a possible remediation agent for microplastics deposited in marine and freshwater ecosystems (Cunha et al. 2019). Information concerning EPS biochemistry and function in chlorophytes is available elsewhere (Xiao and Zheng 2016; LaRoche 2022).

Much of what we know about EPS in green algae remains a major mystery, especially considering that EPS production requires large amounts of the alga's photosynthate and the subcellular secretory machinery. Clearly, detailed glycomics- and proteomics-based analyses will be required to understand the structural and functional roles of these ECM components.

#### CWs: Biochemical diversity and the origin of land plants

The CW represents the most well-studied type of ECM component of plants and green algae. Plant-like CWs are found in the later divergent charophytes and are considered to have been critical in the transition to terrestrial ecosystems and the evolution of land plants. Fibrillar matrix–based CWs as well as CWs with distinct polysaccharide and hydrophobic profiles are also found in the Chlorophyta. Here we present an update on recent discoveries in the CW biology of green algae.

##### The CWs of charophytes

The charophytes consists of 2 main paraphyletic clades: the earlier-diverging KCM-grade (Klebsormidiophyceae, Chlorokybophyceae, and Mesostigmatophyceae) and the later-diverging ZCC-grade (Zygnematophyceae, Coleochaetophyceae, and Charophyceae (de Vries et al. 2016). It is now widely accepted that an ancestor of the Zygnematophyceae emerged onto land approximately 500+ million years ago and gave rise to land plants (Delwiche and Cooper 2015; Hess et al. 2022; Permann et al. 2022). Previous immunochemical and biochemical studies (Domozych et al. 2007; Sørensen et al. 2010, 2011; Popper et al. 2011; Proseus and Boyer 2012; Mikkelsen et al. 2014) illustrated notable similarity in the CW constituents of many charophytes with the primary CWs of many land plants. This includes a scaffolding network of cellulose microfibrils that are embedded in a matrix of pectic polymers, xylan, XyG, mannan, mixed-linkage glucan, and proteins (e.g. arabinogalactan proteins [AGPs], extensin; Zhang et al. 2021). Recently, the sequencing of several charophyte genomes and transcriptomes (Table 1) along with correlative analyses that have synthesized biochemical and ecophysiological data have revealed new information about charophyte CWs/ECM that are refining our understanding of plant evolution, stress physiology, and cell biology (Fitzek et al. 2019; Leebens-Mack et al. 2019; Domozych and Bagdan 2022). Recently, a detailed gene family analysis by Feng et al. (2023) showed that the Zygnematophyceae share all the major enzymes with land plants for CW polysaccharide synthesis, degradation, and modifications. In fact, most of the enzymes for CW innovations that include polysaccharide backbone synthesis were gained more than 700 million years ago. The following represents a summary of highlights concerning recent discoveries of specific components of the CWs of charophytes.

Cellulose, the main load-bearing component of the plant CW, consists of β-D-glucose (Glc) linked by (1–4) glycosidic bonds that assemble to form higher-order fibers, microfibrils, with amorphous and crystalline regions (Nicolas et al. 2022). Cellulose microfibrils are synthesized by a complex of cellulose synthases A (CesA) on the plasma membrane of the cell (Wilson et al. 2021). Cellulose has been found in the late-diverging charophyte clades and in low amounts in the Klebsormidiophyceae and Chlorokybophyceae but not in the Mesostigmatophyceae (Domozych 2012). Fitzek et al. (2019) reported that land plant CesA orthologs exist only in Zygnematophyceae (i.e. the CesA clade) but not in Coleochaetophyceae and Charophyceae of the ZCC clade, nor in the KCM clade. However, cellulose synthaseD-like or CslD-like orthologs have been found in all charophytes analyzed (i.e. the ClsD clade, e.g. Coleochaetophyceae). The CslD-like orthologs were posited to be “charophyte-specific CesAs” that evolved in an ancestral charophyte line. Evolutionary divergence then occurred whereby branches in the CesA clade were only found in ancestral Zygnematophyceae and eventually became the ancestor of land plant CesAs, while another branch became the ancestor of all land plant CslDs (e.g. modern Coleochaetophyceae).

Callose, a β-1,3 glucan, is integral to a wide variety of physiological processes in plants, including rapid responses to abiotic and biotic stress, regulation of plasmodesmata-based transport, the growth of pollen tubes, and cell plate formation during cytokinesis (Schneider et al. 2016). During cytokinesis, callose hydrogels form a flexible and stabilizing matrix during the formation of the cell plate membrane network while also contributing to the radial expansion of the cell plate as it matures into a CW (Sinclair et al. 2022). Callose has been demonstrated in the cytokinetic apparatus of the charophytes *Coleochaete* and *Chara* (Scherp et al. 2001; Delwiche and Cooper 2015). Davis et al. (2020) recently described the role of callose in septum formation during cytokinesis in the unicellular zygnematophyte, *P. margaritaceum*, and posited that this polymer supports the cell plate membrane as it expands inward during new daughter cell pole formation. Putative callose synthase genes were also identified in this study.

Callose has been analyzed in the CW of the charophyte *Klebsormidium*, along with its role in enhancing CW regions experiencing biomechanical stress due to loss of turgor pressure (Herburger and Holzinger 2015, 2016; Steiner et al. 2020). The highly elastic properties of callose enhance/regulate CW flexibility that is necessary for life in habitats with fluctuating water availability, an ancient feature for CW support thought to be critical for dispersal and to have led to the widespread success of these algae.

Hemicelluloses represent a diverse array of CW heteropolysaccharides that possess a ß-(1–>4)–linked backbone and include XyGs, xylans, mannans and glucomannans, and ß-(1–>3,1–>4)-glucans or mixed linked glucans (MLGs) (Scheller and Ulvskov 2010). XyGs function in several ways, including increasing CW rigidity via the tethering of cellulose microfibrils, contributing to CW loosening during expansion, and aggregating soil particles in the rhizosphere around roots (Galloway et al. 2018; Zheng et al. 2018). The β-glucan backbone of XyGs is decorated with a variety of short side-chains interspersed with unsubstituted Glc units at regular intervals (Mikkelsen et al. 2021). Though not originally thought to be found in charophytes, XyGs have since been identified in many charophyte taxa (Sørensen et al. 2010, 2011; Herburger et al. 2018). In a screening of charophyte transcriptomes, Del-Bem (2018) demonstrated that XyG-processing enzymes were found in multiple charophyte taxa. This included the earlier diverging Klebsormidiophyceae (i.e. *K. nitens*) that contained homologs of most of the XyG-related genes first discovered in angiosperms, including all known enzymes required for XyG synthesis. One group of enzymes, the transglycosylases, are CW-remodeling enzymes that cleave off part of the backbone of a polysaccharide (donor) and graft it onto another (acceptor). These molecular rearrangements provide flexibility in the wall that allows for expansion. Transglycosylase activity (e.g. XyG endotransglucosylase (XET)) was demonstrated in the longitudinal CWs of young filaments of *Klebsormidium*, filaments of *Zygnema* (Zygnematophyceae), and the anticlinal and periclinal CWs of parenchymatous *Chara* (Charophyceae) thalli (Herburger et al. 2018). Heterotransglycanases are endotransglycosylases that catalyze transfers between XyGs and MLGs, xylans, or mannans and have also been identified in charophytes (Franková and Fry 2021). Mikkelsen et al. (2021) has also shown that orthologs to the biosynthetic enzymes associated with XyG fucosylation, long thought to be a late evolutionary elaboration, are present in the zygnematophyte *Mesotaenium caldariorum*. It has been postulated that XyGs likely evolved during land colonization by charophytes and contributed not only to cell expansion but also to processing of soils that were key to the conquest of land (Del-Bem 2018).

Mannans constitute another major group of hemicelluloses and contain a backbone of mannose (Man) linked in β-(1-4) configuration or a combination of Glc and Man that can be substituted by α-(1-6)–linked Gal (Moreira and Filho 2008; Scheller and Ulvskov 2010; Voiniciuc 2022). In land plants, mannans function as storage polysaccharides in seedlings and as structural components of the hemicellulose–cellulose network. Mannans have been identified in all groups of charophytes except *Mesostigma* (Sørensen et al. 2011; Permann et al. 2021a), but their location in the charophyte CW architecture and their functions are not yet known. In a recent analysis of transglycosylases, Franková and Fry (2021) noted a major difference in those found in charophytes vs those in land plants. High levels of trans-β-1,4-mannanase acting on mannan substrates were noted that, in turn, suggests that charophytes might prioritize mannan remodeling versus XyG remodeling, which is carried out by transglucanases (Verhage 2021).

Xylans consist of a β-(1-4)–linked D-xylose (Xyl) backbone that can be substituted by L-arabinose (Ara), D-Gal, GlcA, and acetyl groups (Scheller and Ulvskov 2010). Xylans function in growth and development and contribute to the structural integrity of the CW. Like mannans, they are found in most groups of charophytes (Domozych et al. 2010; Sørensen et al. 2011; Hsieh and Harris 2019). Genomic and transcriptomic mining of genes involved in xylan synthesis was performed for the charophyte *K. flaccidum*, which produces a highly substituted ß-1-4-xylan in its CW (Jensen et al. 2018). The protein *K*. *flaccidum* XYLAN SYNTHASE-1 was identified and shown to possess 1,4-β-xylan synthase activity. Also identified were the 1,4-β-xylan xylosyltransferases IRX9 and IRX14 required for xylan synthesis in planta and the glcosylase transfereases GT8, GT43, GT47, and DUF579 (domain of unknown function 579)/GXMT (glucuronoxylan 4-O-methyltransferase-like protein) that represent candidate genes implicated in β-1-4-xylan backbone formation, GlcA addition to the backbone, and subsequent GlcA methylation. *C. brauniia*, a deep-branching, highly diverged form of GT43, was identified as the most likely xylan synthase, providing the first hint that GT43 sequences are enzymatically involved in synthesizing xylan (Nishiyama et al. 2018).

Mixed linkage glucans or MLGs are composed of β-D (1-3) and β-D (1-4)–linked glucosyl residues and have also been identified in charophytes (Sørensen et al. 2011), but little is known about their function.

Pectins are galacturonic acid (GalA)-containing polymers of the plant CW that play critical roles in cell expansion and morphogenesis, ion uptake, and immune signaling pathways (Caffall and Mohnen 2009; Anderson and Keiber 2020; Haas et al. 2021; Cosgrove 2022). Pectins are ancient polymers, and 2 pectin subclasses—homogalacturonan (HG) and rhamnogalacturonan-I—have been found in many charophytes, especially taxa of the ZCC clade (Eder and Lütz-Meindl 2008, 2010; Sørensen et al. 2010, 2011; Domozych et al. 2014; Herburger et al. 2018). HG is a polymer made of (1,4)-GalA that can be substituted with methyl or acetyl groups. Rhamnogalacturonan-I is made of a repeating GalA-rhamnose (Rha) disaccharide that can be substituted with various side chains, such as galactan, arabinan, and type-I arabinogalactan (Caffall and Mohnen 2009). The structural and functional dynamics of charophyte pectins have been studied in detail in several charophytes, including *Chara corallina* and *P. margaritaceum*. In *Chara*, CW expansion encompasses a dynamic pectate cycle that coordinates with Ca^2+^ (calcium)-complexing of HG (Boyer 2016). In *Penium*, the demethylation of recently secreted HG at the central isthmus of the cell and subsequent Ca^2+^-complexing manifest in a bipolar expansion mechanism and the formation of the distinctive HG lattice on the outer CW layer (Domozych et al. 2014; Palacio-Lopez et al. 2020). Screening of the genome and transcriptome of *P. margaritaceum* (Jiao et al. 2020) demonstrated notable gene family expansion for CAZymes, including the classes of glycosyl hydrolase (GH), glycosyl transferase (GT), carbohydrate esterase, polysaccharide lyase, and carbohydrate binding modules. Interestingly, more GTs and polysaccharide lyases were found in *Penium* than in any of the green plant lineages. The precise roles of pectin in charophytes will require further detailed screening, but it is now apparent that these ancient macromolecules have multiple functions in cell expansion, cell differentiation, and the formation of the adhesive middle lamella of multicellular tissues.

Charophytes also possess structural and catalytic proteins in their ECMs. AGPs are one type of non-enzymatic Hyp-rich glycoproteins (Johnson et al. 2003, 2017) that are common CW/ECM components of many photosynthetic eukaryotes, including charophytes (Hervé et al. 2016; Knox 2016; Domozych and Bagdan 2022). These proteins are highly glycosylated (i.e. 90% of a plant's AGP may be carbohydrate), with inclusive arabinogalactan covalently linked to the protein moiety via Hyp (Silva et al. 2020). AGPs have various functions in plant cell expansion, development, and signal transduction across the plasma membrane (Pereira et al. 2015; Lin et al. 2022). In charophytes, putative AGPs have been identified in the CWs of nonconjugating *Spirogyra* filaments, especially in the area of transverse walls (Pfeifer et al. 2022), as well as in the CWs of zygospores of *Spirogyra* and *Mougeotia* (Permann et al. 2021a, 2021b). Biochemical analyses of the *Spirogyra* AGP showed that Ara was present only in small amounts, and the terminating sugars consisted predominantly of terminal and 1,3-linked Rha residues, leading to a new classification of “rhamnogalactan-protein” for this special AGP modification (Pfeifer et al. 2022). Palacio-Lopez et al. (2019) employed immunocytochemical methods to identify AGPs in the ECM surrounding *Spirogyra* rhizoids, *Chlorokybus*, *Coleochaete*, and *Penium*. It was shown that these AGPs were components of various adhesive mechanisms (e.g. cell-cell, cell-substrate).

Extensins are another type of Hyp-rich glycoproteins of plant CWs that act as scaffolds for the deposition of the main CW carbohydrate polymers and also contribute to plant defense (Møller et al. 2017; Castilleux et al. 2021). Extensins have been identified in CWs of a variety of charophytes (Sørensen et al. 2011; Permann et al. 2021a, 2021b). Little detail is known though about their location in the CW or their functions. Bioinformatic analyses reveal that green algae have no classical extensins but have a number of long chimeric extensins (Liu et al. 2016) that are not found in land plants. Glycosyltransferases associated with extension and AGP synthesis have also been identified in green algae (Ulvskov et al. 2013; Mikkelsen et al. 2021).

Using genome-wide transcript expression profiling, Vannerum et al. (2011) demonstrated expansin in the zygnematophyte *Micrasterias denticulata*. A GFP-tagged version of the expansin-resembling protein MdEXP2 localized to the CW and in Golgi-derived vesicles. Overexpression phenotypes altered lobe elongation and caused a loss of growth polarity and planarity. In a study of the transcriptome of *Spirogyra*, Van de Poel et al. (2016) displayed modification of the CW matrix by both expansins and XET/hydrolases.

##### ECMs of earlier diverging charophytes

The ECM of the earlier divergent taxa *M. viride* (Mesostigmatophyceae) and *Chlorokybus atmophyticus* (Chlorokybophyceae) are distinctly different from those found in the Klebsormidiophyceae and later-diverging charophytes (Sørensen et al. 2011). The motile stage of *Mesostigma* produces a covering consisting of layers of intricately shaped scales (see section on prasinophyte scales). The composition of the ECM of the nonmotile phase is unknown. The cells of nonmotile sarcinoid life cycle phase of *Chlorokybus* maintain a thin CW surrounded by an extensive extracellular sheath that contains AGP (Palacio-Lopez et al. 2019), and its motile zoospores are covered by scales. Recent screening of the *Mesostigma* and *Chlorokybus* genomes and transcriptomes (Demko et al. 2019; Liang et al. 2019; Wang et al. 2020; Irisarri et al. 2021) have provided novel insight into ECM dynamics in these 2 algae. *Mesostigma* possesses 81 CAZymes and 20 putative carbohydrate-binding modules (Wang et al. 2020). Three putative cellulose synthase-like (CSL) enzymes (CSLA/CSLC-like) were identified, but CESA was absent. The biosynthesis and transport genes for the 2-keto sugar acid, Kdo, a major component of its scales, was identified (Liang et al. 2019). *Chlorokybus* possesses 100 CAZymes and 22 carbohydrate-binding modules, including 3 CESA/CSLD-like homologues. *Mesostigma* possesses genes involved in CW polymer remodeling, including those involved in mannan and xylan metabolism (e.g. mannanases, mannosidase, and xylosidase), but *Chlorokybus* does not. XyG and xylan-degrading enzymes and pectin lyases are absent from these organisms.

##### Biosynthesis and secretion of the charophyte ECM

Due to their importance in the evolution of plants, charophytes and their subcellular machinery involved in ECM/CW synthesis and secretion have garnered much interest. The structure and dynamics of the endomembrane system and cytoskeletal network during CW/cell expansion and EPS production have been focal points of many of these studies, and detailed reviews may be found in Lütz-Meindl (2016) and Domozych and Bagdan (2022). The development of the CW/ECM during cytokinesis, including furrowing and phragmoplast/cell plate formation, the formation of the plasmodesmata, and morphogenetic events leading to multicellular thallus, has also attracted significant interest (Pickett-Heaps 1975; Buschmann and Zachgo 2016; Brunkard and Zambryski 2017; de Vries and Archibald 2018; Nishiyama et al. 2018; Buschmann 2020; Davis et al. 2020).

##### The CWs of chlorophytes

The Chlorophyta represent the largest and most diverse group of green algae. Several classes of chlorophytes have been identified (Leliaert 2019), most recently based on data from phylogenomic studies (Gulbrandsen et al. 2021; Li et al. 2021; Yamashita et al. 2021; Table 1). However, the very limited screening of the genomes/transcriptomes of vast assortment of chlorophyte taxa leaves many fundamental questions unresolved (Bachy et al. 2022). Many chlorophytes have been studied in detail, and several have become valuable model organisms for various biological investigations (e.g. *Chlamydomonas*, *Volvox*; Matt and Umen 2016; Craig et al. 2021). Others are recognized as keystone primary producers of the planet's oceans (e.g. *Micromonas*; Demory et al. 2019), and several are harvested and used for the medical, food, and biofuel applications (e.g. *Ulva*; Roleda and Heesch 2021). Chlorophyte phenotypes range widely from minute unicells to macroscopic unicellular siphons to large seaweeds. The ECMs of chlorophytes are very diverse. An excellent and thorough review of the structure and chemistry of their CWs and other extracellular components is presented in Baudelet et al. (2017). Three general types of ECMs have been recognized in the chlorophytes: Group 1 contains organisms with ECMs comprised of scaly or thecal coverings that contain deoxy-5-*O*-methyl-*manno*-2-octulosonic acid and deoxy-*lyxo*-2-heptulosaric acid (Becker et al. 1994; see section on prasinophyte scales); Group 2 taxa have glucan, mannan, and chitin-containing CWs with alagenan and some have crystalline glycoprotein CWs (see section on glycoprotein walls); and Group 3 organisms have glucan and mannan CWs that also contain sulfated or pyruvylated polysaccharides.

### Recent highlights of select chlorophyte taxa

The Trebouxiophyceae consist of a diverse array of phenotypes commonly found in soil, and subaerial habitats and as the phycobionts of lichens. Several inclusive taxa have become important models for research and as economically important resources.

***Chlorella*:** The unicellular chlorophyte *Chlorella* has been a popular organism in algal/plant physiology (e.g. the alga used by Melvin Calvin and co-workers in elucidating carbon fixation in photosynthesis; Nickelsen 2017). Today, *Chlorella* and related genera (e.g. *Nannochloropsis gaditana*) have shown much promise for yielding large CW-based biomass for conversion to biofuels (Rodrigues and de Silva Bon 2011; Taleb et al. 2015) as well as being employed in various medical and food supplement technologies (Bito et al. 2020). Species of this alga often have a bilayer or unilayer CW (Weber et al. 2022). The outermost layer of the bilayer wall often possesses a trilaminar structure and contains the highly resistant compound sporopllenin. Cellulose is found in the inner CW layer, and chitin or chitin-like polymers have been identified in the CW as well (Weber et al. 2022).

***Botryococcus:*** One of more unique chlorophytes is the colonial *Botryococcus*. A total 86% of its dry weight can consist of long-chain hydrocarbons, including tri-terpine-based oils (e.g. botryococcenes), alkadienes, and alkatrienes. This and other features have brought *Botryococcus* into the limelight of applied technologies such as biofuels, food additives (e.g. carotenoids), cosmetics, nutraceuticals, feed stock, and antibiotic nanoparticle production (Cheng et al. 2019a, 2019b; Love 2022). The botryococcenes are produced in fenestrated cortical ER that directly delivers these hydrocarbons to the plasma membrane ,where they are deposited externally, exuded across the CW and into the ECM (Weiss et al. 2012). *Botryococcus*'s ECM is structurally complex (Wang et al. 2022) and is made of 3 components: 1) a fibrillar, β-1, 4- and/or β-1, 3-glucan–containing CW; 2) an intracolonial space that consists of a cross-linked hydrocarbon network that is permeated with liquid hydrocarbons; and 3) a retaining wall that contains of a polysaccharide sheath of fibrils that sequester the ECM liquid hydrocarbons (Weiss et al. 2012). The hydrocarbon-ECM complex fills the interstices between cells and may act to stabilize the colony and facilitate gas exchange in the colony interior. The fibrils consist of 97% carbohydrate containing significant amounts of arabinose (42%) and Gal (39%).

***Trebouxia, Coccomyxa:*** *Trebouxia* and *Coccomyxa* are 2 unicellular chlorophytes that may be free-living in terrestrial habitats or live as phycobionts of lichens (Centeno et al. 2016; González-Hourcade et al. 2020, 2021; Spribille et al. 2020, 2022). Lichens and their green microalgae are considered poikilohydric organisms, that is, they do not actively regulate their water content and must depend on the availability of water in their habitats. Many lichens live in habitats of extreme abiotic stress, especially with fluctuations in water availability, and recent work has shown that *Trebouxia* and *Coccomyxa* CW structural and chemical modulations are critical for their survival in the lichen complex (Baudelet et al. 2017). These lichen algae have flexible CWs that fold as the protoplast shrinks during dehydration and expand during rehydration. This flexibility is believed to prevent the mechanical stress occurring during dehydration in cells. β-3-Linked rhamnogalactofuranan, KOH-soluble β-glucans, and sulfated polysaccharides have been isolated from *Trebouxia* and *Coccomyxa*, and details of their CW chemistry and biochemical remodeling under stress can be obtained in González-Hourcade et al. (2021, 2020) and Gasulla et al. (2021).

### Ulvophytes

The ulvophytes (Ulvophyceae) constitute a wide array of mostly macroscopic marine chlorophytes (i.e. green seaweeds) and some subaerial and freshwater taxa. Ulvophyte phenotypes range from unicells to macroscopic sheet-like thalli to giant-celled multinucleate siphonous and siphoncladous forms (Cocquyt et al. 2010). *Ulva*, a common and widely distributed green coastline seaweed, has been the focal point for many of ECM studies of ulvophytes (Lahaye and Robic 2007; Wichard et al. 2015; De Clerck et al. 2018; Wahlström et al. 2020a, 2020b; Kloareg et al. 2021; Wichard 2023). This seaweed grows in large amounts, has been harvested for a variety of human uses (Smetacek and Zingone 2013; Simon et al. 2022), and is also the cause of “green tides” (i.e. blooms caused by high nutrient run off in coastal zones; Hiraoka 2021). Four groups of polysaccharides have been characterized in the ECM of *Ulva*: semicrystalline cellulose, water-soluble ulvans, XyG, and a glucuronan (Kloareg et al. 2021). Ulvan, is a high–molecular weight (660,000–760,000 g/mol) sulfated polysaccharide that makes up to 30% of the alga's biomass and is composed of repeating units of sulfated disaccharides with Rha, Xyl, GlcA, and iduronic acid as the main building blocks (Kidgell et al. 2019, 2021; Wahlström et al. 2020a, 2020b; Kloareg et al. 2021). These include β-d-GlcA (1 → 4)-α-l-Rha-3-sulfate and α-l-iduronic acid (1 → 4)-α-l-Rha-3-sulfate. The iduronic acid or GlcA components may instead be a Xyl unit (sulfated or nonsulfated) forming the characteristic monomers β-d-Xyl (1 → 4)-α-l-Rha-3-sulfate and β-d-Xyl-2-sulfate(1 → 4)-α-l-Rha-3-sulfate. Sulfated polysaccharides like ulvan most likely serve as physiological adaptations to the high ionic strength of the saltwater habitats and contribute to moisture retention that enhances desiccation resistance. They also are important in cell-cell interactions, adhesion mechanisms, and as defense against pathogens (Ciancia et al. 2020). The development of an ulvan-specific monoclonal antibody has aided immunocytochemical studies (Rydahl et al. 2017). Sulfated polysaccharides from *Ulva* and other marine algae are gaining significant interest in that they are being screened for anti-cancer, anti-angiogenic, antioxidant, anti-inflammatory, immunostimulatory, hydrogel, and antiviral activities (Sulastri et al. 2021; López-Hortas et al. 2021; Meinita et al. 2022; Negreanu-Pirjol et al. 2022). Detailed comparative reviews of the complex sulfated polysaccharides found in other ulvophyte groups, including the Ulotrichales, the giant multinucleate (coenocytic) cells of the siphonous Bryopsidales and Dasycladales, and siphonocladous freshwater Cladophorales, are provided elsewhere (Fernández et al. 2015; Arata et al. 2017; Ciancia et al. 2020). Recently, pectins have been identified in *Ulva* (Holzinger et al. 2015), and the first identification of O-acetylation of pectin in green algae has been described in Bothwell et al. 2022. These results demonstrate that many CW polymers arose in green algae well before the emergence of land plants.

Cellulose is also a component of the CW of ulvophytes (Thygesen et al. 2021). Using atomic force microscopy, cellulose microfibril cross-linking and interactions with CW matrix components were demonstrated in *Ventricaria ventricosa* (Eslick et al. 2014). In another atomic force microscopy–based analysis of the CW of *Valonia utricularis*, a CW matrix component containing *N*-acetylgalactosamine was shown to be involved in the maintenance of the CW integrity through bonding of the neighboring CW layers (Mine and Sekida 2018). Linear β-1,4-mannans replace cellulose as the dominant structural polymer in some ulvophycean taxa, such as the green seaweed *Codium vermilara* (Fernández et al. 2015). This algal mannan is mostly fibrillar, with 2-linked sulfate on 23% of Man units resulting in partially soluble chains that could function in maintaining amorphous CW regions.

AGPs have also been identified in *Ulva* (Přerovská et al. 2021), and amino acid analysis showed a similarity between these and land plant AGPs. However, AGPs from *Ulva* revealed unique glycosylation patterns with relatively low amounts of arabinose and Gal but the presence of 3-O-methyl-hexose. These results likely show a specialized adaptation to the marine environment and add new insight to the evolution of CWs in ulvophytes and green algae in general.

Screening of the genomes of *Ulva* (*Ulva mutabilis*, De Clerck et al. 2018; *Ulva compressa*, Osorio et al. 2022) and *Caulerpa lentillifera* (Arimoto et al. 2019) have also contributed to our understanding of the ECM of ulvophytes. The *Ulva* species’ genomes contain multiple cellulose synthases, hydroxyprolyl O-arabinosyl transferases, glucomannan-mannosyl transferase, UDP-glucosamine transferase, UDP-rhamnose transporters, UDP-uronic acid transporters, pectin lyase, extensins, and expansin (Osorio et al. 2022). Interestingly, genes encoding for collagens and elastins have been detected in these 2 species as well. Comparative analyses of the biochemistry and genetics of ulvophytes and other seaweeds are provided in Kloareg et al. (2021) and Shao and Duan (2022).

#### The ECM of the Prasinodermophyta

The Prasinodermophyta is a newly recognized phylum of green algae thought to have diverged before the split of Chlorophyta and Charophyta 1 billion years ago. At present, the CW/ECM (Jouenne et al. 2011) has not been biochemically or structurally characterized. Mining of the Prasioderma coloniale genome revealed that genes responsible for the biosynthesis and remodeling of the major components of the plant primary CW (e.g. cellulose, mannans, xylans, XyGs) were not present (Li et al. 2020).

## Concluding remarks

Over the last decade, an infusion of new information concerning the CWs/ECMs of green algae has significantly refined our understanding of green plant evolution; the CW and its role in expansion, development, and ecophysiology; and the applied uses of these cell coverings in multiple industries (see Advancements). Yet today, only a very small percentage of green algae, their CWs/ECM, and their biosynthetic/secretory pathways have been investigated in detail. Future studies will be needed to thoroughly elucidate the structure and function of both known polymers and the unique components of green algal ECMs. This information will yield critical insight into such areas as the architectural design and functional dynamics of specific polymers in the green plant CW, ECM modulations that occur during abiotic stress (e.g. low water stress, UV exposure), the functions of the complex EPS gels and hydrophobic molecules in cell coverings, as well as identify novel model organisms for investigating basic green plant life processes (Outstanding Questions Box). As important, these studies will directly contribute to our understanding of the causes and controls of algal blooms (e.g. green tides caused by *Ulva*), provide new ways to control atmospheric CO_2_ through mass algal growth, and reveal further uses of CW/ECM materials in food, medicine, and the development of clean biofuels.

## Data Availability

All data is available upon request from the author, DSD.
